# 42956 Patterns and impact of long-term glucocorticoid use on RA patients at risk for major adverse cardiac events

**DOI:** 10.1017/cts.2021.760

**Published:** 2021-03-30

**Authors:** Beth I. Wallace, Yuqing Gao, Punyasha Roul, Shirley Cohen-Mekelberg, Bryant England, Ted Mikuls, Daniel J. Clauw, Rodney Hayward, Akbar K. Waljee

**Affiliations:** 1 Center for Clinical Management Research, VA Ann Arbor Healthcare Center and Division of Rheumatology, Department of Internal Medicine, Michigan Medicine; 2 Center for Clinical Management Research, VA Ann Arbor Healthcare Center; 3 Division of Rheumatology and Immunology, Department of Internal Medicine, University of Nebraska Medical Center; 4 Center for Clinical Management Research, VA Ann Arbor Healthcare Center and Division of Gastroenterology, Department of Internal Medicine, Michigan Medicine; 5 Department of Anesthesiology, Michigan Medicine; 6 Division of General Medicine, Department of Internal Medicine, Michigan Medicine

## Abstract

ABSTRACT IMPACT: Glucocorticoid steroids are commonly used despite known dose-dependent cardiovascular toxicity, yet little is known about a) how patients with other cardiovascular risk factors use glucocorticoids, and b) how risks of glucocorticoid treatment might vary depending on a patient’s baseline cardiovascular risk. OBJECTIVES/GOALS: Up to one-third of RA patients use long-term glucocorticoids (GCs) despite a known, dose-dependent association with increased risk of major adverse cardiovascular events (MACE). We aim to evaluate patterns of GC use among RA patients with other MACE risk factors (i.e. diabetes, smoking), and examine how GC use may potentiate these risk factors. METHODS/STUDY POPULATION: We used claims data from Veterans Health Administration to identify 6,090 RA patients with ≥1 rheumatology clinic visit during 2013-2017. We used logistic regression to evaluate associations between incident MACE between 2013-2018, recent long-term GC use, and 5 MACE risk factors: hypertension, diabetes, hyperlipidemia, smoking, and prior MACE. We included two-way interaction terms between GC use and each risk factor. We used a claims-based algorithm to define MACE as any of acute MI, ischemic stroke, TIA, sudden death, or coronary revascularization, between index date and 12/31/2018. We defined index date as first rheumatology visit after meeting RA diagnostic criteria, and recent long-term GC use as ≥90 days’ supply dispensed over 2 years prior to index date. RESULTS/ANTICIPATED RESULTS: Among 2,884 eligible patients,1,553 (54%) had MACE risk factors, and 97 (3%) had prior MACE (Table 1). Overall, 16% of patients recently used long-term GC, compared to 17% of patients with MACE risk factors, and 22% of patients with prior MACE. Incident MACE occurred in 308 (11%) patients, 24% of whom had recent long-term GC use. Recent long-term GC use was independently associated with increased incident MACE (Table 2). While no interaction term was statistically significant overall, differences in odds of incident MACE were seen across levels of recent GC use for several risk factors, particularly diabetes (OR 2.10, 95% CI [0.93-4.77]), tobacco use (OR 2.88, 95% CI [1.16-7.14]) and prior MACE (OR 2.41, 95% CI [0.73-7.95]). DISCUSSION/SIGNIFICANCE OF FINDINGS: Long-term GC use is common among RA patients with MACE risk factors. In this cohort, 25% of patients with incident MACE had recently used long-term GC. Long-term GC use may potentiate effects of comorbidities like diabetes and smoking, disproportionately increasing MACE risk in certain patients.

